# microRNAs and long non‐coding RNAs as biomarkers for polycystic ovary syndrome

**DOI:** 10.1111/jcmm.17139

**Published:** 2022-01-06

**Authors:** Mona Tamaddon, Mostafa Azimzadeh, Seyed Mohammad Tavangar

**Affiliations:** ^1^ Chronic Diseases Research Center, Endocrinology and Metabolism Population Sciences Institute Tehran University of Medical Sciences Tehran Iran; ^2^ Stem Cell Biology Research Center Yazd Reproductive Sciences Institute, Shahid Sadoughi University of Medical Sciences Yazd Iran; ^3^ Medical Nanotechnology & Tissue Engineering Research Center Yazd Reproductive Sciences Institute, Shahid Sadoughi University of Medical Sciences Yazd Iran; ^4^ Department of Medical Biotechnology, School of Medicine Shahid Sadoughi University of Medical Sciences Yazd Iran; ^5^ Department of Pathology, Dr. Shariati Hospital Tehran University of Medical Sciences Tehran Iran

**Keywords:** long non‐coding RNA, microRNAs, ovary, polycystic ovary syndrome

## Abstract

Polycystic ovary syndrome (PCOS) is known as the most common metabolic/endocrine disorder among women of reproductive age. Its complicated causality assessment and diagnostic emphasized the role of non‐coding regulatory RNAs as molecular biomarkers in studying, diagnosing and even as therapeutics of PCOS. This review discusses a comparative summary of research into microRNAs (miRNAs) and long non‐coding RNAs (lncRNAs) that are molecularly or statistically related to PCOS. We categorize the literature in terms of centering on either miRNAs or lncRNAs and discuss the combinatory studies and promising ideas as well. Additionally, we compare the pros and cons of the prominent research methodologies used for each of the abovementioned research themes and discuss how errors can be stopped from propagation by selecting correct methodologies for future research. Finally, it can be concluded that research into miRNAs and lncRNAs has the potential for identifying functional networks of regulation with multiple mRNAs (and hence, functional proteins). This new understanding may eventually afford clinicians to control the molecular course of the pathogenesis better. With further research, RNA (with statistical significance and present in the blood) may be used as biomarkers for the disease, and more possibilities for RNA therapy agents can be identified.

## 1 INTRODUCTION

Polycystic ovary syndrome (PCOS) is known as the most common endocrine/metabolic disorder in women of reproductive age, where it is reported that PCOS affects 5 to 26 percent of women (based on the applied diagnostic criteria) around the world.^1^, ^2^ PCOS was found to be associated with different diseases such as metabolic syndrome, type 2 diabetes, hypertension, cardiovascular diseases and even ovulatory infertility.^3^, ^4^ There are four distinguished subtypes; inflammatory, hidden cause and finally, pill‐induced PCOS and insulin‐resistant (the most prevalent type).^5^, ^6^ Although the complete etiology of the syndrome remains unclear, the genetic, epigenetic, environmental factors and lifestyle have been associated with PCOS causality. In clinical and/or biochemical settings, diagnosis is based on whether the patient shows no less than two out of three main symptoms, including cysts in the ovaries, high androgen levels and irregular periods. Despite the controversial opinions of current diagnostic criteria of PCOS, an expert opinion from the ultrasound, pelvic exam and blood tests can confirm the diagnosis.^7^, ^8^ Blood tests for PCOS are mainly based on hormone levels and endocrine function, and there is a growing clinical need for biomarkers that are both sensitive and specific enough for PCOS diagnosis.^9^ Biomarker research can also enable clinicians to achieve earlier diagnosis, molecularly subtype the disease in the clinic and cast light on the underlying molecular mechanisms of the disease and its derivatives, mainly cancer and diabetes.^10^

In modern medicine, the multilateral relationship between the RNAs and different proteins determines how molecular disease conditions change functions. We know that RNAs have regulatory functions in the way of protein production. It may follow that the disorders in protein levels that lead to many diseases could have first happened at the RNA level. Earlier changes could be diagnosed in the RNA level as an early diagnosis method for diseases with genetic traces. Recent developments in RNA immunotherapies, RNA vaccines and a vast field of RNA biomarkers are examples of their increasing role in modern medicine. The central role of RNAs in the transcriptome network makes them promising candidates as molecularly significant biomarkers of disease.^11^, ^12^

**FIGURE 1:**
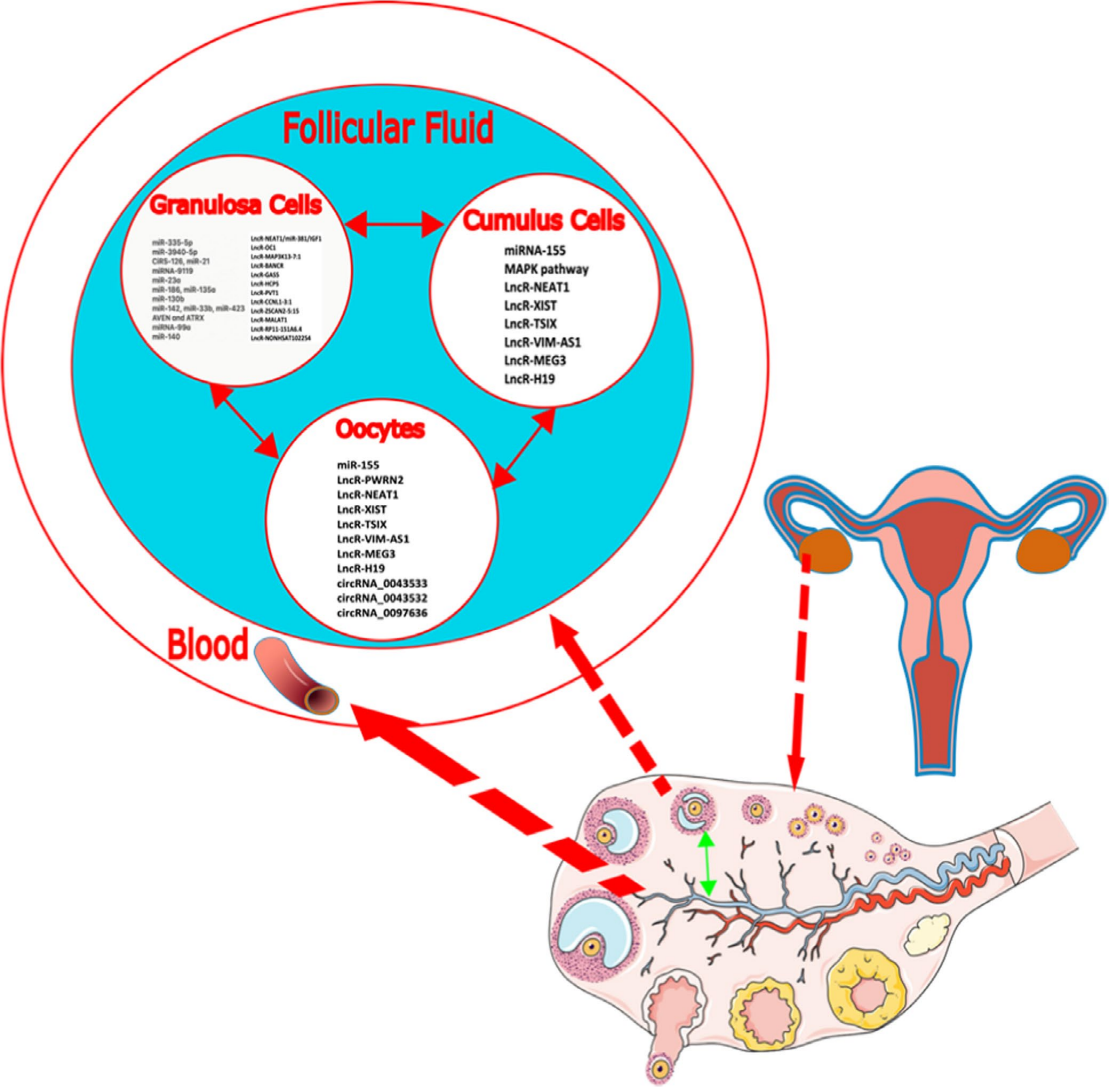
Biomarkers for PCOS, a schematic for how molecularly disease condition affects biomolecule levels, from cells to intercellular connections, such as gap junctions between cumulus and granulosa cells. Cross‐talks between ovarian tissue cells and oocytes and exosomes mediating the intercellular networks, all exposed to follicular fluid and hence blood serum environment

RNAs vary greatly in roles, size, conformation and sequence. Non‐coding RNAs (ncRNAs) (not translated into proteins) with complex regulatory functions have gene and protein regulatory functions. Two main groups are miRNAs with about 22 nucleotides and long non‐coding RNAs (lncRNA).^13^, ^14^ Both have roles in the cell's physiology, especially in post‐transcriptional regulations and could have a signature on every single disease or any biological malfunction. Some present in blood could be easily accessible through different body fluid samples.^15^, ^16^ Hence, their role has been discovered as a diagnostic biomarker for many diseases such as PCOS^17^ and cancer.^18^ Through advances in sequencing technology and systems biology over the past decade, their application in discovering the molecular mechanism of complex diseases such as PCOS has become higher, leading to the discovery of new precise and feasible biomarkers.^19^, ^20^ Figure 1 represents the biomarkers for PCOS analysis.

In this review, we first overview the prominent research groups working on miRNAs and PCOS, and then those working on lncRNAs. We follow with a discussion of combinatory and creative studies into the molecular basis of PCOS. We discuss how each research approach can be helpful on its own or when combined or integrated with other available methodologies. We also discuss the innovative methods and approaches that allowed leading research groups to gather key findings and share our insights into how these findings may determine future research into the molecular mechanisms and biomarkers for PCOS. This article also includes descriptive finding data for the found RNA biomarkers.

## 2 miRNAs AND lncRNAs AS BIOMARKERS FOR PCOS

### 2.1 miRNAs and lncRNAs

The functional molecular role of RNAs has been observed in many key processes, such as their enzymatic role in protein translation at ribosomes.^21^ Any other types of RNA, other than coding RNAs, that translate to proteins are called non‐coding RNAs, which can either be a direct transcript of a gene or a messenger RNA intron. miRNAs are short non‐coding single‐stranded RNAs (22–23 nucleotides (nt) long), with an important role in gene regulatory processes.^22^ They act like core elements in the transcriptome, mediating correlations between different genes representing RNAs or proteins. They can bind to various RNAs and biological sites in order to regulate cell functions.^23^ Besides intra‐cellular functions, inter‐cellular relationships are also heavily dependent on miRNAs, and hence their presence is abundant in body fluids, and they have become a hotspot in biomarker research, particularly for PCOS.^11^, ^12^

Long non‐coding RNAs, on the other hand, are normally more than 200nt long and can have either linear or circular conformation. lncRNAs are core to many cellular functions such as gene regulation, transcription, chromatin modification and epigenetic regulation.^24^ According to a recent discovery, there are many more lncRNAs in line to be discovered or functionally understood. With the presence in exosomes and being a means of cellular crosstalk overall, they are good candidates for being a biomarker in body fluids, just like miRNAs.^25^ A wide range of RNA therapies are in the line for coming years, with applications in personalized medicine.^26^ The technology to synthesize desired nucleotide sequences *in vitro* has achieved recent worldwide prominence in synthesizing an RNA‐based COVID‐19 vaccine.

### 2.2 miRNAs as biomarkers for PCOS

One of the major symptoms of PCOS is the presence of numerous small cysts in the ovaries. One method used to diagnose PCOS in the clinic is internal sonography of the ovaries.^5^, ^27^ Sonography, however, normally might be uncomfortable for the patient and cannot distinguish between different disorders that come with cysts. Blood tests to determine testosterone levels are another common clinical approach to diagnose PCOS. However, high levels of hormones such as testosterone are only observed in the late stages of PCOS, where the patient is at an elevated risk for developing a diabetic response. Genetically, early diagnosis with hormone levels is nearly impossible.

In general, there are two main approaches to validate a reliable biomarker for the disease. One is to use the potential of sequencing and blotting to differentially investigate a pool of possible RNAs and proteins in two populations of healthy and PCOS affected cells and patient blood samples or follicular fluid. From this pool, the most significant biomarkers are selected. The second is to investigate the molecular mechanism and biological significance of a known ncRNA biomarker for the syndrome.

As far as finding new biomarkers is concerned, there is diversity but promise in the results. Che et al^28^ identified a pool of 55 differentially expressed miRNAs in PCOS conditions, from which the most significantly differentiated (miR‐27a‐5p) had a strong correlation with the incidence of cancer in patients with PCOS. In studies similar to this one, the researchers compile a set of differential data with various statistical significances. Key meta‐analysis has been performed on these data sets by Deswal et al.^29^ They used an initial group of 79 miRNAs from 21 studies, reported to be differentially expressed, and only three of which were reported in more than three studies. After the meta‐analysis, they reported miR‐29a‐5p and miR‐320 as significant biomarkers for PCOS. They moved further in their meta‐analysis, and for the three most significant markers found, they performed a genetic and functional analysis, as shown in Figure 3. These key meta‐analyses determine the significance of results by evaluating their replicability in the literature, preventing the possible scientific error from propagation by eliminating the unrepeated biomarkers. This kind of noise cancellation is vital in research, especially when we will use the found biomarkers for further genetic and functional analysis.

Several statistically significant miRNAs have already been identified as candidates for a clinical PCOS biomarker. The most statistically significant miRNAs included 381‐3p, 29a‐5p, 93, 320, 3188, 612, 509‐3p, 547‐3p, −5p, 20‐3p and −5pn (for a complete list, refer to Figure 1, Table 1 and Table 2). In order to further verify the scientific significance of each biomolecule, researchers must investigate its molecular role or path of efficacy. This is the approach that most groups have taken when researching PCOS at the molecular level.

**TABLE 1:** Summarizing the findings for miRNAs regarding polycystic ovary syndrome

Team	miRNAs	Findings and notes
Che et al.^28^	miR−27a−5p	‐ Role in endometrial cancer cell migration and invasion, correlation with cancer ‐ From blood serum
Yao et al.^32^	miR−335‐5p	‐ Decreases granulosa cell proliferation, targets SGK3 gene (Serum/Glucocorticoid Regulated Kinase Family Member 3) ‐ Samples from follicular fluid
Udesen et al.^39^	miR−122, miR−223, miR−29a	‐ Metformin decreases the three in PCOS patients
Gao et al.^34^	miR−3940‐5p	‐ Increases granulosa cell proliferation and follicular development ‐ Targets KCNA5 gene (Potassium Voltage‐Gated Channel Subfamily A Member 5)
Lu et al.^51^	CiRS−126, miR−21	‐ The circular RNA inhibits granulosa cell, targets the miR−21‐PDCD4‐ROS axis in the PCOS model
Deswal et al.^29^	miR−29a−5p, miR−320, miRNA−93	‐ A systematic review and meta‐analysis
Jiang et al.^40^	miR−146a−5p, miR−126–3p, miR−20b−5p, miR−106a−5p	‐ Descriptive analysis of PCOS miRNAs, increased miR−146a−5p and miR−126–3p with decreased miR−20b−5p, miR−106a−5p
Hocaoglu et al.^55^	miR−16‐5p, miR−155‐5p	‐ Increased miR−16‐5p expression is associated with PCOS in pregnancy. Also, decreased miR−155‐5p expression was found in relation with gestational diabetes mellitus (GDM)
Sharma et al.^35^	Notable Descriptive Study	‐ A database of genes, diseases, pathways and networks associated with polycystic ovary syndrome
Arancio et al.^56^	miR−155	‐ Biomarker for monitoring the estroprogestinic treatment ‐ On serum miRNAs
Ebrahimi et al.^57^	miR−146a	‐ (PCOS) associated with the CC genotype of miR−146a gene variation
Dehghan et al.^58^	miRNA−155	‐ Regulates cumulus cell function, oocyte maturation and blastocyst formation
Ghasemi et al.^59^	Polymorphism in H19 Gene	‐ Relation with PCOS in Iranian population
Yu et al.^60^	Polymorphisms in the RAB5B gene	‐ Relation with PCOS in Chinese population
Diaz et al.^61^	miR−451a	‐ Low circulating levels of miR−451a in PCOS ‐ In serum
Ding et al.^62^	miRNA‑9119	‐ Regulates cell viability of granulosa *via* mediating Dicer expression
McAllister et al.^63^	miRNA−130b−3p	‐ Mediates DENND1A variant2 expression and androgen biosynthesis
Cirillo et al.^64^	miR−146a, miR−155, miR−320, miR−370, miR−486	‐ miRNAs regulating insulin sensitivity are affected in PCOS. Also, associated with markers of inflammation.
Butler et al.^65^	miR−1260a, miR−18b−5p, miR−424‐5p, and miR let−7b−3p	‐ Increased microRNA levels in PCOS in people without insulin resistance (a relatively less molecularly identified sub‐group) ‐ The expressed miRNAs were associated with the inflammatory pathways involving TNF and IL6. Circulating miRNAs were identified, using qPCR.
Sorensen et al.^66^	miR−1290, miR−20a−5p, miR−139‐3p, miR−433‐3p, and miR−361‐5p	‐ Hyperandrogenism and metabolic syndrome are associated with changes in serum‐derived microRNAs in PCOS
Lionet et al.^67^	miRNA−27b	‐ Circulating and adipose tissue miRNAs, strong correlation with PCOS
Rooda et al.^68^	8 miRNAs	‐ Cellular, extracellular and extracellular vesicular miRNA of pre‐ovulatory follicles
Wang et al.^45^	miR−486‐5p, miR−4651	‐ miR−486‐5p may be implicated in follicular development in PCOS by targeting PRELID2. ‐ Also, miR−4651 may be involved in inflammation via leukocyte transendothelial migration
Luo et al.^69^	miR−23a	‐ Regulatory function of miR−23a in granulosa cell apoptosis
Song et al.^70^	miR−186, miR−135a	‐ Altered miR−186 and miR−135a contribute to granulosa cell dysfunction by targeting ESR2
Xue et al.^71^	miR−29a, miR−132, miR−151 and miR−155	‐ All the four differentially expressed are involved in androgen metabolism or function
Xia et al.^72^	miR−155	‐ miR−155 is high‐expressed in PCOS and promotes cell proliferation and migration by targeting PDCD4 ‐ On KGN cells
Hou et al.^73^	miR‑ 3188, miR‑3135b	‐ The expression level of hsa‑miR‑3135b was significantly correlated with the number of oocytes retrieved, the fertilization rate and the cleavage rate
Jiang et al.^40^	miR−130b	‐ miR−130b regulates gap junctional intercellular communication through connexin 43 ‐ On granulosa cells from PCOS patients
Li et al.^74^	miR−142, miR−33b, miR−423	‐ Dysregulated miR−142, −33b, and −423 in granulosa 2 cells target TGFBR1
O’Doherty et al.^75^	A set of prominent miRNAs	‐ Expression of granulosa cell microRNAs, AVEN and ATRX are associated with human blastocyst development
Nanda et al.^76^	miRNA−24, miRNA−29a and miRNA−502‐3p	‐ Correlation with biochemical parameters related to PCOS and insulin resistance
Geng et al.^77^	miRNA−99a	‐ miRNA−99a regulates proliferation and apoptosis of human granulosa cells via targeting IGF−1R in PCOS
Nearmeen et al.^78^	miRNA−320	‐ miRNA−320 expression level and its target gene endothelin−1 correlated with PCOS
Xiong et al.^79^	miR−140	‐ miR−140 targets RAP2A to enable the proliferation of insulin‐treated ovarian granulosa cells
Butler et al.^80^	Descriptive on PCOS miRNAs in pic 2019	‐ In follicular fluid
Pourteymour Fard Tabrizi et al.^81^	miR−27a, miR−301a, miR−130b	‐ miR−27a and miR−301a had a significant increase but the miR−130b expression level decreased in the patient group ‐ From The circulating plasma

**TABLE 2:** Summarizing the finding for lncRNAs regarding polycystic ovary syndrome

Team	Main LncRNAs	Notes and findings
Butler et al. 2019^82^	AC005332.6 MALAT1 AC009404.1 MIR181A1HG PSMG3‐AS1	‐ No lncRNA correlated with anti‐mullerian hormone (AMH) levels, insulin resistance (HOMA‐IR) or the free androgen index (FAI). ‐ LncRNAs differ between anovulatory PCOS and control women in the follicular phase of the menstrual cycle
Tan et al.^83^	LncRNA SRA1	‐ LncRNA SRA1 gene single‐nucleotide polymorphism correlated to polycystic ovary syndrome
Jiao et al.^84^		‐ LncRNA and mRNA profiles in follicular fluid from mature and immature ovarian follicles of healthy women and women with PCOS, construction of the mRNA/lncRNA network ‐ Good example of systematic transcriptome‐wide analysis
Fawzy et al.^85^	Circ‐LncRNAs: H19, GAS5	‐ Also, associated with type 2 diabetes
Huang et al.^44^	LncRNA‐PWRN2	‐ Construction of a lncRNA‐PWRN2‐ ceRNA network suggests its potential roles in oocyte nuclear maturation in PCOS patients
Ma et al.^38^	LINC00667, H19, AC073172.1	‐ Construction of PCOS related lncRNA‐mRNA network ‐ Three main PCOS related lncRNAs were involved in the NF‐kB signaling pathway, inflammatory, apoptotic and immune‐related processes
Huang et al.^37^	Exosomal circLDLR	‐ CircLDLR increased miR−1294 expression and inhibited CYP19A1 expression in recipient cells ‐ Related to ovarian steroidogenesis, aldosterone synthesis and secretion, and Jak‐STAT signaling. ‐ From follicular fluid
Bouckenheimer et al.^86^	LncRNAs: NEAT1, XIST, TSIX, VIM‐AS1, MEG3 and H19	‐ Differential lncRNA expression profiles in human oocytes and cumulus cells, ‐ MII oocyte lncRNAs could be involved in chromatin remodeling, cell pluripotency and in driving early embryonic development.
Jiang et al.^87^	LncRNA‐HOTAIR	‐ Downregulated lncRNA‐HOTAIR alleviates PCOS by reducing expression of insulin‐like growth factor 1 via miRNA−130a, on ovarian tissues of rat
Zhen et al.^88^	LncRNA NEAT1/miR−381/IGF1	‐ Downregulated lncRNA NEAT1 upregulates microRNA−381 to induce proliferation ‐ and repress apoptosis of ovarian granulosa cells in PCOS rat, ‐ Through inhibiting IGF1 expression. ‐ LncRNA NEAT1 acted as a competing endogenous RNA to adsorb miR−381, and IGF1 was verified to be a direct target gene of miR−381.
Wu et al.^89^	Lnc‐OC1	‐ Its downregulation associated with PCOS, in granulosa cells
Lin et al.^90^	LncRNA GAS5	‐ Downregulation of lncRNA‐GAS5 may contribute to insulin resistance in PCOS patients ‐ From serum
Liu et al.^91^	LncRNA‐Xist	‐ Xist downregulation may be involved in PCOS and is correlated with adverse pregnant outcomes in PCOS ‐ From serum
Wang et al.^92^	lncRNA‐H19	‐ high‐throughput lncRNA sequencing of follicular fluid exosomes in non‐PCOS infertility patients and PCOS infertility patients ‐ In exosomes from follicular fluid ‐ lncRNA‐H19 represented the largest node and was predicted to have the potential to interact with 15 target miRNAs
Zeng et al.^30^	lncRNAs (KLF3‐AS1, WWC2‐AS, and MAPKAPK5‐AS1) miRNAs(miR−382)	‐ Construction of a drug molecule and RNA network ‐ Based on co‐expression and ceRNA network analyses
Zhang et al.^93^	lncRNA CD36–005	‐ Identification of mRNAs related to endometrium function regulated by lncRNA CD36–005 in rat endometrial stromal cells ‐ Providing a list of potential target mRNA genes of CD36–005 in endometrial stromal cells and laid a foundation for further studies on the molecular function and mechanism of CD36–005 in the endometrium helping to unfold the PCOS
Geng et al.^94^	lncRNA‐MAP3K13‐7:1	‐ Inhibits ovarian granulosa cells proliferation in PCOS via DNMT1 downregulation ‐ In KGN cells ‐ lnc‐MAP3K13‐7:1 overexpression resulted in cell cycle arrest in the G0/G1 phase, as well as the molecular inhibition and genetic silencing of DNMT1.
Yang et al.^95^	LncRNA‐BANCR	‐ Role in PCOS by promoting apoptosis in granulosa cells ‐ From cells of IVF patients
Wang et al.^54^	LncRNA‐GAS5	‐ LncRNA‐GAS5 is upregulated in polycystic ovary syndrome and regulates cell apoptosis and the expression of IL−6 in granulosa cells ‐ From blood plasma
Sun et al.^53^	lncRNA‐H19	‐ lncRNA H19 acts as a ceRNA to regulate the expression of CTGF by targeting miR−19b in PCOS ‐ On KGN cell line ‐ H19 could promote cell proliferation and decrease cell apoptosis
Chen et al.^96^	LncRNA‐HCP5	‐ LncRNA‐HCP5 promotes cell proliferation and inhibits apoptosis via miR−27a−3p/IGF−1 axis ‐ On human granulosa‐like tumor cell line KGN
Guo et al.^97^	LncRNA‐HOTAIRM1	‐ LncRNA‐HOTAIRM1/miR−433‐5p/PIK3CD function as a ceRNA network to encourage the development of PCOS
Han et al.^98^	LncRNA‐LET	‐ LncRNA‐LET inhibits cell viability, migration and EMT while induces apoptosis by up‐regulation of TIMP2 ‐ On KGN cell line
Liu et al.^99^	lncRNA PVT1	‐ lncRNA‐PVT1/MicroRNA−17‐5p/PTEN axis regulates secretion of E2 and P4, proliferation, and apoptosis of ovarian granulosa cells
Butler et al. 2020^100^	LINC01539, AC095350.1, LINC00616	‐ LncRNA Expression in Non‐obese PCOS and weight matched controls ‐ Differed in serum
Qin et al.^101^	LncRNA‐H19	‐ LncRNA‐H19 is associated with PCOS in Chinese women ‐ From peripheral blood leukocytes
Huang et al.^47^	Lnc‐CCNL1‐3:1	‐ lnc‐CCNL1‐3:1 promotes granulosa cell apoptosis and suppresses glucose uptake in PCOS ‐ From human luteinized granulosa cells(hLGCs) derived from women
Zhao et al.^102^	LINC−01572:28	‐ LINC−01572:28 inhibits granulosa cell growth via a decrease in p27 in PCOS
Sang et al.^103^	LncRNA‐NEAT1	‐ LncRNA‐NEAT1 drives the development of PCOS *via* sponging multiple miRNAs
Youssef et al.^104^	LncRNA steroid receptor activator (SRA)	‐ LncRNA‐SRA has positive correlation with hirsutism, obesity, testosterone, and insulin resistance in PCOS patients. ‐ LncRNA‐SRA may be a mediator in the pathogenesis of both metabolic and hormonal syndromes.
Li et al.^105^	LncRNA‐TUG1	‐ Molecular mechanisms for LncRNA‐TUG1 in PCOS
Che et al.^106^	lnc‐ZSCAN2‐5:15	‐ Promotes follicular fluid androgen excess in PCOS patients via aromatase inhibition. ‐ In granulosa cells derived from PCOS and non‐PCOS women
Liu et al.^17^	Lacking to provide p‐values for the greatly changed markers	‐ Using human granulosa cells (GCs) and the KGN cell line.
Zhang et al.^36^	LncRNA‐MALAT1	‐ lncRNA‐MALAT1 is involved in the pathogenesis of PCOS through TGFβ signaling in granulosa cells ‐ A nice biomolecule for possible future RNA therapy, repeated in literature
Wang et al.^107^	LncRNA‐H19	‐ Metformin and sitagliptin combination therapy is effective for PCOS with insulin resistance through upregulation of lncRNA‐H19. To summarize, co‐treatment induced H19 expression via suppressing the PI3K/AKT‐DNMT1 pathway.
Ma et al.^50^	circRNA_0043533, circRNA_0043532, circRNA_0097636	‐ Serum testosterone (T) level positively correlated with the expression of circRNA_0043533 and circRNA_0097636 in the PCOS group ‐ Dysregulated circRNAs were possibly involved in cell cycle, oocyte meiosis, progesterone‐mediated oocyte maturation, the FOXO signaling pathway, phosphatidylinositol signaling and glycerophospholipid metabolism
Zhao et al.^108^	LncRNA *RP11*‐*151A6*.*4*	‐ *RP11*‐*151A6*.*4* was identified as a hub lncRNA based on IRLMN and WGCNA and was highly expressed in ovarian granulosa cells, skeletal muscle, and subcutaneous and omental adipose tissues of patients with insulin resistance ‐ Relationships with: insulin resistance, androgen excess, and adipose dysfunction in PCOS patients
Jin et al.^109^	LncRNA‐NONHSAT102254	‐ In ovarian granulosa cells from women with PCOS with or without hyperandrogenism ‐ dysregulated lncRNA in PCOS have a regulatory role in mitochondrial function via interacting with transcription factors such as YY1 and SIX5.
Gao et al.^110^	LINC00477	‐ The LINC00477/miR−128 axis promotes the progression of PCOS, via regulating ovarian granulosa cell proliferation and apoptosis. ‐ From serum of patients and model.
Li et al.^111^	LncRNA‐SRA	‐ Up‐regulation LncRNA‐SRA promotes cell growth, inhibits cell apoptosis, and induces secretion of estradiol and progesterone. ‐ From ovarian granular cells of mice. ‐ Elevated LncRNA stimulated cell growth, changed distribution of cell cycle phases with increase of Cyclins B, E, and D1, and inhibited cell apoptosis with increment of bcl2 and decrease of bax, cleaved‐caspase 3, and cleaved‐PARP.
Li et al.^112^	lncRNA‐SRLR	‐ Upregulation of the lncRNA‐SRLR regulates cell apoptosis and increases levels of interleukin−6 (IL−6). ‐ Also, in renal cell carcinoma, the lncRNA‐SRLR upregulates IL−6.
Fu et al.^46^	Expression profiles of mRNA and lncRNA	‐ LncRNA–miRNA–mRNA network was constructed ‐ On rat ovaries through deep sequencing
Zhao et al.^31^	circ_0023942	‐ circ_0023942 inhibits the proliferation of human ovarian granulosa cell

**FIGURE 2:**
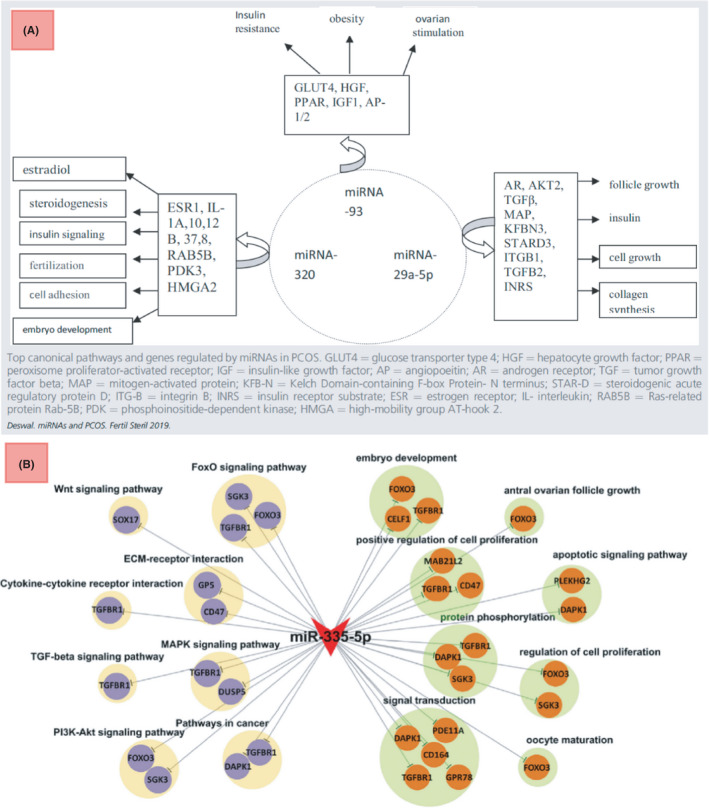
(A) Deswal et al. functional analysis for the three most prominent miRNA biomarkers^29^ (B) Yao et al. mir‐335‐mRNA network for GO and KEGG analyses centered on the discovered differentially expressed miRNA, miR‐335‐5P^32^

Deswal et al. have demonstrated that functional analysis can be effective, even without discovering a new biomarker for the disease. They performed a bioinformatics analysis of recent findings in the field. After selecting three significant miRNAs from the existing literature, they investigated the pathways and functions related to them. All three miRNA biomarkers were strongly correlated with the insulin cycle (Figure 2A), adding further evidence for a meaningful correlation between insulin metabolism and PCOS conditions. This correlation cannot easily be called causation, and even if causation is to be determined, it could be in either direction between diabetic and PCOS conditions.

Research into PCOS gene ontology and pathway encyclopaedia analysis has also integrated functional analysis.^30^, ^31^ Yao et al. identified a functional network for a verified biomarker.^32^ After discovering the statistical significance of the miRNA‐335‐5p in the follicular fluid of patients with PCOS, a model of the possible underlying mechanism was tested. They observed a decrease in KGN cell line proliferation by inhibiting the activation of the AKT and mTOR pathways. They also reported SGK3 activation and proliferation inhibition in granulosa cells derived from patients with respect to healthy people. They moved further to perform gene ontology and pathway analysis using the Kyoto Encyclopedia for Genes and Genomes for the miRNA‐335‐5p. High affinity in the correlation between the miRNA and signal transduction mechanisms was expected. However, relationships with cancer pathways and pathways such as WNT in Kyoto Encyclopedia of Genes and Genomes (KEGG) analysis corroborates the correlation with positive regulation of cell proliferation suggested by Gene Ontology^32^ (Figure 2B). These results were not individualistic and were corroborated with many other groups in sharing common functions such as cell proliferation and follicular development.^33^, ^34^

Recent publications report correlations between various RNAs and signaling networks, working towards identifying possible complex protein‐RNA matrices.^30^, ^35^ Although we currently have far more knowledge of protein functions in the physiology of the cell than we do of RNA functions, these matrices would be a great asset to discover more about RNA functions as well. Some groups have been focusing on finding biomarkers by identifying RNA correlation with signaling networks. Mao et al. used parallel Western blotting and RT‐PCR methods to identify a correlative response of a biological vector (miR‐126‐5p/miR‐29a‐5p/klotho/insulin‐IGF‐1/Wnt/Akt) to PCOS.^33^ Using knockout mice, they confirmed the genetic effectiveness of the Klotho pathway for the syndrome. However, the significance of other elements of the biological vector, such as WNT or the miRNAs, as a genetic basis for the syndrome, remains precisely verified. Nevertheless, Mao et al. identified a useful set of biomarkers for PCOS. Their results indicated that miR‐126‐5p and miR‐29a‐5p along with the insulin growth factor 1 (IGF‐1R) and Wnt family member1 (Wnt1) are downregulated, where the Klotho protein expression is higher in the mice model and in patients with PCOS. Overall, their research demonstrates how investigations into the correlative responses of miRNAs and proteins for PCOS can enhance our knowledge of the biological vectors involved and identify new biomarkers.

Classically, we thought of signaling networks as solid protein expression rules. To recapitulate, we specifically investigated the proteins when studying their relationships and called them signaling networks. From that standpoint, numerous studies correlated miRNAs and lncRNAs to key factors in some classical signaling networks. The idea is only to correlate some types of RNAs to some key proteins, according to their significance in classical signaling networks. For example, LncRNA‐MALAT1 has been correlated to TGFβ signaling,^36^ exosomal circLDLR to Jak‐STAT^37^ and the triple (LINC00667, H19 and AC073172.1) to the NF‐kB signaling pathway.^38^ However, there is a need to move farther to redesign the classical view and build a modern one; to engage many types of RNAs and proteins as they are present together in the cell in order to figure out modern transcriptome networks consisting of various types of RNAs as well as proteins altogether. In this manner, databases can be great guides to minimize the costs of research. Noticeably, there has been insightful research performed here as a guide for us to move forward. For instance, some of their outputs are depicted in Figures 2 and 4. As shown, these are perfect examples of how to construct modern networks using databases and molecular meta‐analysis. To restate, Figure 2 depicts the outputs of groups investigating huge biomolecule networks with insight towards proteins and their entailed physiological properties in databases, while Figure 4 depicts more insightful and modern network constructions using various elements such as proteins, RNAs or even drugs. In the light of these types of methodologies in research, there seems to be enough light to pass the way towards knowing more of the complex relationship between RNAs and proteins as prominent functional biomolecules. However, this has to be performed through more experimental investigations and big data build‐up and analysis.

As drugs are normally agents that oppose the molecular mechanisms of diseases, studying how drugs can affect miRNA levels can be beneficial in selecting the differentially expressed ones for investigation. Testing the effects of common drugs for PCOS on the levels of different miRNAs can help detect new potential biomarkers for the disorder. The found miRNAs can be useful for immunology research of the disease related to the new field of miRNA vaccines (available now due to Covid‐19 global vaccine action). Not surprisingly, drugs can alter the levels of miRNA in PCOS conditions as well. Udesen et al.^39^ found metformin to decrease three miRNAs, potentiating them as biomarkers for PCOS condition classifications. They identified miR‐122, miR‐223 and miR‐29a to be significantly decreased by metformin in PCOS conditions. The three miRNAs can now be potentials to be incorporated in biological vectors and complexes in molecular PCOS research, both for the immunology or biogenesis of the disorder. In the study by Udesen et al and similar studies, the correlation between miRNAs and any given drug is figured out only in PCOS conditions. In order to determine the molecular role of metformin in the human body, the effects on blood miRNA levels can be investigated in different conditions such as diabetes or on the population with both conditions. This promises better statistical knowledge on the drug's overall effect on body function, and the biological vectors consisting of different RNAs and proteins between a set of disorders can be determined.

With miRNAs compromising the greatest share of RNAs in exosomes, exosomal miRNAs are a sub‐group with demonstrated scientific significance for PCOS diagnosis,^28^, ^40^ with the recently uncovered role of exosomes in creating complex signaling networks between cells of various compartments in the human body. PCOS is a condition with multi‐organ symptoms. In other words, cells in various parts of the body with vastly different protein profiles get affected while the overall miRNA expression in a body liquid sample is determined. Hence, miRNAs associated with extracellular signaling, more common among cells than those mostly associated with intracellular networks, seem to be better candidates for a biomarker for the disorder in body liquids. On the other side, exosomes are one of the main ways for signal transport between cells. Hence, exosomal miRNAs are key elements to look at in this field. However, regarding the exosomes, there are various types of RNA present in them, including circular and overall lncRNAs. Many groups focused on exosomal RNA subtypes and their role in PCOS—Wang et al. identified circRNAs in exosomes of the follicular fluid.^41^ With the role of circRNAs and exosomes in intercellular communication, RNAs present in follicular fluid contain important information on how cumulus, granulosa and other cell types of the ovaries surrounding the oocytes interconnect and how the disease is molecularly developed in this network. Hu et al. did the same for the small RNAs.^42^ Wang et al. performed a bioinformatics analysis of differential lncRNAs and found a central role for lncRNA‐H19 in PCOS. Altogether, research has verified the molecular role of multiple types of RNA present in exosomes of the follicular fluid. Exosomes are also present in blood serum, but relating serum exosomes to a particular disorder will require extensive additional scientific research. There are significant difficulties in separating exosomes from other free biomolecules such as RNAs in the sample. The abundance of exosomes from numerous tissues in the serum reduces the specificity of exosomal conformation to a particular syndrome such as PCOS or a specifically affected tissue such as the ovarian tissue. Therefore, although exosomes in the blood may seem to be more clinically useful for PCOS, exosomes from the follicular fluid contain much more specific and useful information for identifying PCOS in the laboratory.

Numerous technologies recently boosted the use of RNAs in modern medicine. Modern sequencing, mRNA synthesis and modern genome editing are the most prominent. Recent progress in sequencing has enabled us to perform rapid descriptive transcriptome‐wide analysis.^20^ Improvements in mathematical and computational biology and the evolvement of systems biology approaches have contributed to mining the transcriptome data. RNA synthesis technology has also enabled researchers to fabricate desired nucleotide sequences *in vitro*, which can make various therapeutic and diagnostic agents. The COVID‐19 pandemic is also increasing the speed of the process of RNA synthesis in diagnostics, vaccine and therapeutic industries. These methods and the complete set of bioinformatics enabled many groups, as summarized in Table 1, to perform research on miRNAs and PCOS.

### 2.3 lncRNAs as biomarkers for PCOS

Efforts for better insight into the competing endogenous(ce)RNA networks affecting PCOS have also led to higher knowledge of the molecular networks and functions in the endocrine and female reproductive system overall. Overall, the literature indicates that lncRNAs play a key role in making up endogenous networks of bilaterally effective relationships between proteins, nucleic acids and various other types of RNAs (mainly mRNAs and miRNAs).^24^ As a biomarker such as miRNAs, lncRNAs will be significant in accordance with their central regulatory role in the transcriptome.^43^ Various groups have exploited polymerase chain reaction (PCR), sequencing technology and computational biology techniques to investigate different possible lncRNAs as biomarkers with diversity in statistical significance. For instance, the study by Huang et al.^44^ on the incorporation of bioinformatics exhibits the molecular significance of each of the biomolecules as biomarker candidates for PCOS. Technically, there are parameters with regard to the significance of the found biomarker. Among the most prominent are the *p*‐value or overall statistical significance, the fold of change in condition, specificity of the change to the condition, sensitivity of the change to special symptoms and the molecular role of the biomolecule.

**FIGURE 3:**
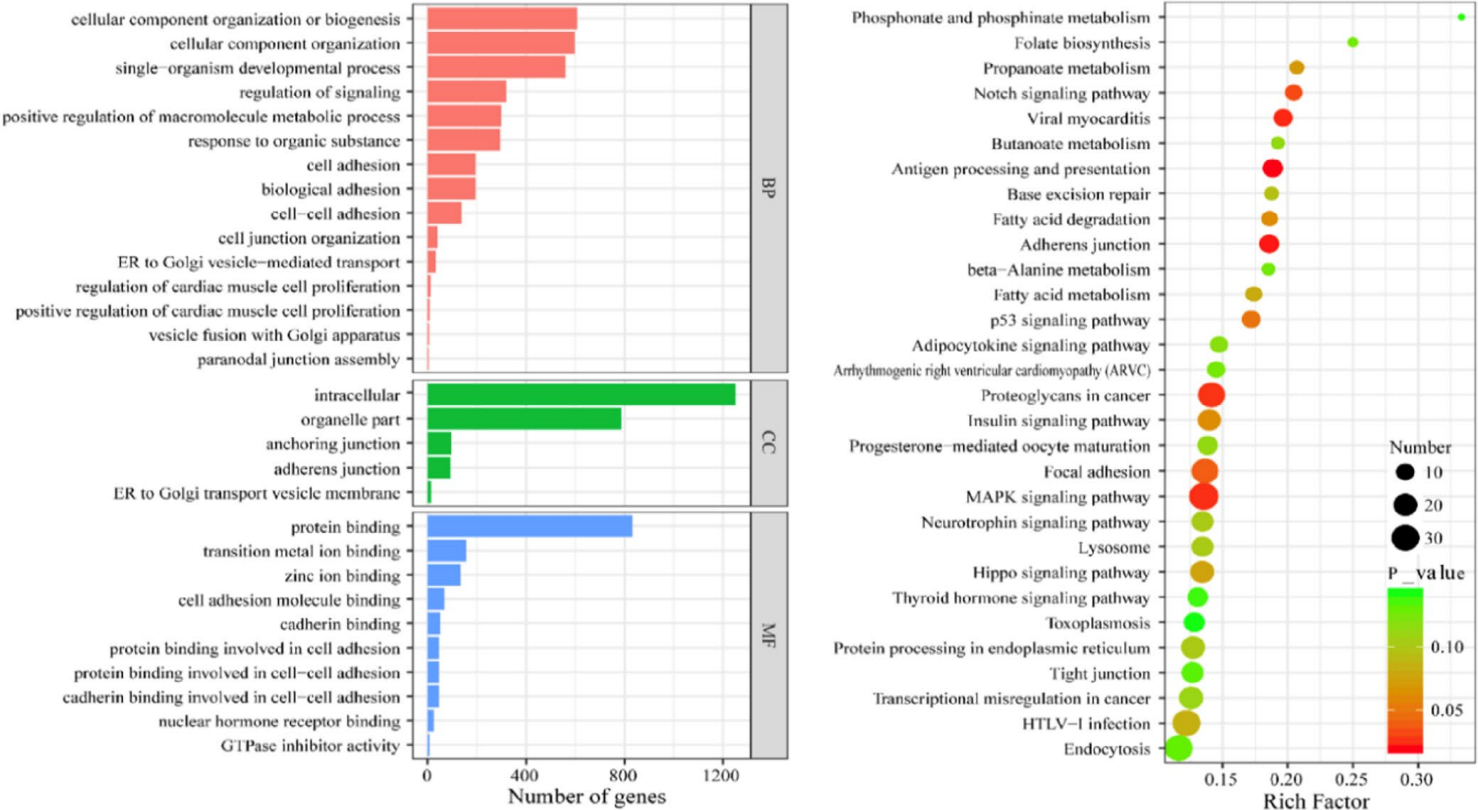
Wang et al. to explore the functions and pathways related to the differentially expressed lncRNAs, they performed GO and KEGG pathway analyses by using the DAVID bioinformatics tool (version 6.8)^92^

Multiple groups have exploited the Gene Ontology (GO) and pathway analysis with Kyoto Encyclopedia of Genes and Genomes (KEGG) to mine out substantial relationships between their discovered RNA biomarkers for PCOS and important related biological functions.^30^, ^31^ Wang et al reported a deep bioinformatic analysis of the pathways and functions of multiple biomarkers (i.e. differentially expressed lncRNAs)^45^ (Figure 3). Fu et al investigated the KEGG pathway analysis of their set of differentially expressed mRNAs.^46^ They discovered that the differentially expressed mRNAs are associated with several specific signaling pathways, including transcriptional misregulation in cancer, IR, biosynthesis of steroid hormone, PPAR signaling pathway, cell adhesion molecules, leukocyte transendothelial migration, the interaction of cytokine/its receptor, AMPK signaling pathway and finally autoimmune thyroid disease. Analysis of the combination of these pathways and the related genes with GO analysis (as was done by Wang et al.) gives better insight into the related functions to be used in therapeutics, as well as verification of the strength of the discovered relationships.

**FIGURE 4:**
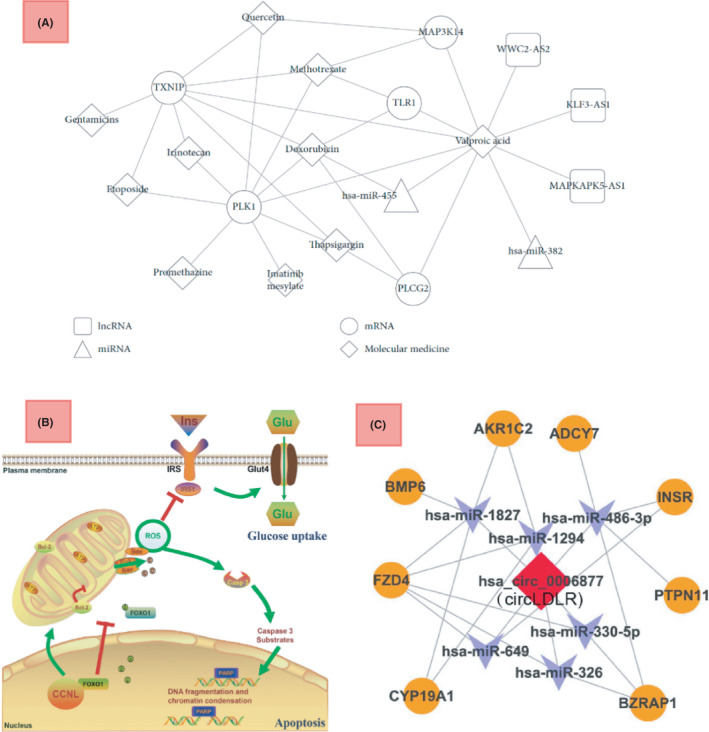
(A) Zeng et al. construction of a drug‐biomolecule network for PCOS,^30^ (B) Huang et al. Schematic diagram for lnc‐CCNL1‐3:1 function in women with PCOS, Upregulation of CCNL interacts with the transcription factor FOXO1, impairs the mitochondria function, promotes cell apoptosis and reduces glucose uptake in women with PCOS.^47^ (C) Huang et al. ceRNA network for circLDLR^37^

Zeng et al.^30^ contributed to the efforts for therapeutic innovation in PCOS. With deep insight into bioinformatics approaches, they have constructed an RNA‐drug network based on the found differentially expressed miRNAs and lncRNAs. Logically, RNAs act as mathematical and biological mediators to figure out molecular correlations among different drugs. Correlations can guide future research for finding out causations, that is, evident molecular mechanisms leading the stream of the syndrome. This type of research is also highly applicable for finding out functional roles for the RNA biomarkers. To recapitulate, with many of the functional efficacy features of the drugs being known, strong correlations between a biomarker and a set of drugs sharing efficacy on a body function will be a hint for a possible strong correlation between the biomarker and the function. The main beauty of their research is that they facilitate the physicians with a mathematical tool, which is the diagram in Figure 4, to build the therapeutic strategy. However, as they also acknowledged in the article, further clinical and model studies should be in queue to be performed in order to verify the differential RNAs' substantiality, the strength of the RNA‐drug relationships and involved molecular mechanisms. One final important note to make about their research is that the correlations for lncRNAs have only been observed for valporic acid, and for miRNAs only with valporic acid and doxorubicin. In contrast, all the tested drugs show strong correlations with the mRNAs. However, it is not scientifically clear that this difference would carry a biological meaning, since the selection set of different types of RNAs is not uniformly covering all the possible RNAs of the three types in the whole transcriptome.

Huang et al.^47^ on the other hand, has focused more on the underlying molecular mechanism very recently. With their beautiful schematic, as observed in Figure 4, they exhibit how an increase in glucose uptake occurs in granulosa cells after the increase in lncRNA‐CCNL1‐3:1. This type of research is beneficial to verify the substantiality of the discovered biomarkers. More importantly, this methodology excludes false correlation‐causation conclusions derived in statistical analysis of the transcriptome network. To recapitulate, however, the statistical significance of a biomarker would work for the diagnostical side, and it would not ensure its effectiveness for therapeutics until we investigate the underlying molecular mechanism. Furthermore, combining this type of work with the RNA‐drug network methodology incorporated by Zeng et al. as explained above, would be a good way for finding suitable therapeutic biological complexes.

Circular RNAs are the type of long RNAs with a covalently closed‐loop shape and may form coding or non‐coding genes. Hence, they can be both gene information carriers for proteins as well as gene regulators.^48^ Predictably, their interaction with other types of RNA and their role in the transcriptome for a wide range of illnesses has been of interest in recent research, including PCOS. For instance, Huang et al. have compromised a ceRNA network based on their discovered differentially expressed circular RNA, the circLDLR (from the parental transcript for low‐density lipoprotein receptor), for a deeper analysis.^37^ They analyzed co‐expression features of the circLDLR/miR‐1294/CYP19A1 ceRNA network in granulosa cells of PCOS. With the network verified to be differentially expressed, they further investigated functional genes related to their circular RNA. For this, they first computationally found out most probably sponged miRNAs to the circLDLR and then they mined out the affected targets for them (Figure 4). A very important note to make here is the error that the computational analysis may carry in relating miRNAs that are theoretically and sequentially correlated but not functionally correlated at all. To restate, there should be scientific evidence for the possibility of functional correlation for each of the miRNAs with the circLDLR in granulosa cells, first *in vitro* and then in *vivo*. There are also other studies focused on circular RNAs in PCOS. Both Che et al. and Ma et al., have performed a descriptive study on circRNAs present in cumulus cells, which is of high prominence in PCOS research, given the role of cumulus cells in oocyte maturation and the role of circRNAs in cellular interconnection.^49^, ^50^ Lu et al. also found out that the circular RNA CiRS‐126 inhibits granulosa cells by targeting miRNA‐21, suggesting again the circular RNAs' role in regulatory processes as also Zhao et al. did find a similar effect for circ_0023942^31^, ^51^ in reducing granulosa cell proliferation. Also, Deng et al.^52^ is another team that verified circPUM1 efficacy on PCOS by sponging to miR‐760.

As far as the type of biopsy for the biomarker is concerned, there are numerous groups working on blood serum, follicular fluid or cells of the ovarian tissue. Also, in order to trigger the attention on new biomarkers for the disease, studies on cell lines can also be favorable, especially when the line is correlated to a special condition of the disease, like that for KGN as performed by many groups.^17^, ^31^, ^44^, ^47^, ^53^, ^54^ Blood serum seems feasible for clinical diagnostics, and statistically significant biomarkers in the blood are not few. It is important to note that a differentially present biomolecule in the blood components is a very significant biomarker for the syndrome and not necessarily an early stage indicator. Hence, in order to take a deeper swim into the vast sea of interconnected biomolecules in the underlying molecular mechanism of the disease, tissue and cell analyse are of essence, especially with the type of biology involved in follicular development and, as a result, in the normal function of the ovaries that is heavily dependent on cellular interconnection. To enlighten, cumulus cells are a cluster of closely related granulosa cells around the oocyte, and their function is related to the maturation and fertilization of the oocyte. Hence, studies like those by Bouckenheimer et al. or Jiang et al. cast light on how cells of the ovaries interconnect RNA‐wise help in the way of unfolding the molecular basis of the syndrome and, overall, women's reproductive system. As, for instance, Jiang et al found that miR‐130b intercellular communication by gap junctional *via* connexin 43 in granulosa cells and Bouckenheimer et al. showed that oocyte lncRNAs could have roles in chromatin remodeling, cell pluripotency and also in the early development of the embryo. They did so by analyzing the differential lncRNA expression profiles in human oocytes and cumulus cells. A manner pretty widespread among groups, as a list of prominent studies regarding lncRNAs and PCOS with similar methods, is available in Table 2.

## 3 CONCLUSION

Polycystic ovary syndrome (PCOS) is a disorder in which women of reproductive age suffer, causing disorder in levels of hormones. Having direct relation with infertility issues and causing long‐term health damage by mechanism (like increasing the chance for diabetes), PCOS comes with an excessive need for early and precise diagnosis. The correlation between hormone levels and PCOS conditions indicates that endocrine cellular function in epithelial levels has been significantly affected. This opens up the possibility for developing diagnostic biomarkers and miRNAs and small non‐coding RNAs for use in a clinical setting. In this study, we review recent research into how the syndrome exploits miRNAs and lncRNAs in its molecular path and whether this presence and effect makes RNA a biomarker for the diagnosis of PCOS. We also compared the type and methodology of research for prominent groups and their pros and cons. We make the following suggestions for future research: First, there is a need for more meta‐analysis to summarize the statistical data of all the sequencing data from various groups. That is essential in eliminating the errors inherent in sequencing and any bias in selecting the biomolecules of interest in different groups. With rapid sequencing technology, it is not too optimistic to expect reliable RNA biomarkers in clinical use worldwide in the near future. Second, a great deal of research is needed to find more combinatory RNA networks formed of different types of known RNAs in combination with proteins and functional signaling networks. This is where a great insight for PCOS therapeutics is observed. With more unveiling of the molecular mechanism of PCOS, there will be significant core RNAs discovered as the best candidates for RNA immunotherapy development.

## CONFLICT OF INTEREST

The authors declare that there is no conflict of interest.

## AUTHOR CONTRIBUTIONS

**Mona Tamaddon:** Conceptualization (equal); Visualization (equal); Writing – original draft (equal). **Mostafa Azimzadeh:** Writing – original draft (equal). **Seyed Mohammad Tavangar:** Conceptualization (equal); Project administration (lead); Supervision (lead); Writing – review & editing (lead).

## Data Availability

Data sharing not applicable—no new data generated for the review article.

## References

[1] Ye W , Xie T , Song Y , Zhou L . The role of androgen and its related signals in PCOS. J Cell Mol Med. 2021;25(4):1825‐1837. doi:10.1111/jcmm.16205 33369146 PMC7882969

[2] Chang S , Dunaif A . Diagnosis of polycystic ovary syndrome: which criteria to use and when? Endocrinol Metab Clin North Am. 2021;50(1):11‐23. doi:10.1016/j.ecl.2020.10.002 33518179 PMC7860982

[3] Luo Y , Cui C , Han X , Wang Q , Zhang C . The role of miRNAs in polycystic ovary syndrome with insulin resistance. J Assist Reprod Genet. 2021;38(2):289‐304. doi:10.1007/s10815-020-02019-7 33405004 PMC7884539

[4] Abraham Gnanadass S , Divakar Prabhu Y , Valsala GA . Association of metabolic and inflammatory markers with polycystic ovarian syndrome (PCOS): an update. Arch Gynecol Obstet. 2021;303(3):631‐643. doi:10.1007/s00404-020-05951-2 33439300

[5] Sirmans SM , Pate KA . Epidemiology, diagnosis, and management of polycystic ovary syndrome. Clin Epidemiol. 2013;6(1):1‐13. doi:10.2147/clep.s37559 24379699 PMC3872139

[6] Dapas M , Lin FTJ , Nadkarni GN , et al. Distinct subtypes of polycystic ovary syndrome with novel genetic associations: An unsupervised, phenotypic clustering analysis. PLoS Med. 2020;17(6):e1003132. doi:10.1371/journal.pmed.1003132 32574161 PMC7310679

[7] Sensale S , Ramshani Z , Senapati S , Chang HC . Universal Features of Non‐equilibrium Ionic Currents through Perm‐Selective Membranes: Gating by Charged Nanoparticles/Macromolecules for Robust Biosensing Applications. J Phys Chem B. 2021;125(7):1906‐1915. doi:10.1021/acs.jpcb.0c09916 33410691

[8] Oguz SH , Yildiz BO . An update on contraception in polycystic ovary syndrome. Endocrinol Metab. 2021;36(2):296‐311. doi:10.3803/EnM.2021.958 PMC8090477 33853290

[9] Burks HR , Wild RA . Diagnostic criteria and epidemiology of PCOS. In: Polycystic Ovary Syndrome: Current and Emerging Concepts. Vol 9781461483. Springer New York; 2014:3‐10. doi:10.1007/978-1-4614-8394-6_1

[10] Khatami F , Larijani B , Tavangar SM . The presence of tumor extrachomosomal circular DNA (ecDNA) as a component of liquid biopsy in blood. Med Hypotheses. 2018;114:5‐7. doi:10.1016/j.mehy.2018.02.018 29602465

[11] Shabani N , Razaviyan J , Paryan M , et al. Evaluation of miRNAs expression in medullary thyroid carcinoma tissue samples: miR‐34a and miR‐144 as promising overexpressed markers in MTC. Hum Pathol. 2018;79:212‐221. doi:10.1016/j.humpath.2018.05.019 29885402

[12] Shabani N , Sheikholeslami S , Paryan M , et al. An investigation on the expression of miRNAs including miR‐144 and miR‐34a in plasma samples of RET‐positive and RET‐negative medullar thyroid carcinoma patients. J Cell Physiol. 2020;235(2):1366‐1373. doi:10.1002/jcp.29055 31297834

[13] Moradi‐Chaleshtori M , Hashemi SM , Soudi S , Bandehpour M , Mohammadi‐Yeganeh S . Tumor‐derived exosomal microRNAs and proteins as modulators of macrophage function. J Cell Physiol. 2019;234(6):7970‐7982. doi:10.1002/jcp.27552 30378104

[14] Torabi S , Tamaddon M , Asadolahi M , et al. miR‐455‐5p downregulation promotes inflammation pathways in the relapse phase of relapsing‐remitting multiple sclerosis disease. Immunogenetics. 2019;71(2):87‐95. doi:10.1007/s00251-018-1087-x 30310937

[15] Khatami F , Larijani B , Nasiri S , Tavangar SM . Liquid biopsy as a minimally invasive source of thyroid cancer genetic and epigenetic alterations. Int J Mol Cell Med. 2019;8(2):19‐29. doi:10.22088//IIJMCM.BUMS.8.2.19 32351906 PMC7175608

[16] Khatami F , Tavangar SM . Liquid biopsy in thyroid cancer: New insight. Int J Hematol Stem Cell Res. 2018;12(3):234‐247. doi:https://pubmed.ncbi.nlm.nih.gov/30595827 PMC6305265 30595827

[17] Liu YD , Li Y , Feng SX , et al. Long noncoding RNAs: Potential regulators involved in the pathogenesis of polycystic ovary syndrome. Endocrinology. 2017;158(11):3890‐3899. doi:10.1210/en.2017-00605 28938484

[18] Tamaddon M , Shokri G , Hosseini Rad SMA , Rad I , Emami Razavi À , Kouhkan F . Involved microRNAs in alternative polyadenylation intervene in breast cancer via regulation of cleavage factor “CFIm25”. Sci Rep. 2020;10(1):11608. doi:10.1038/s41598-020-68406-3 32665581 PMC7360588

[19] Fiocchi C , Iliopoulos D . IBD systems biology is here to stay. Inflamm Bowel Dis. 2021;27(6):760‐770. doi:10.1093/ibd/izaa343 33438720

[20] Goodwin S , McPherson JD , McCombie WR . Coming of age: Ten years of next‐generation sequencing technologies. Nat Rev Genet. 2016;17(6):333‐351. doi:10.1038/nrg.2016.49 27184599 PMC10373632

[21] Cooper TA , Wan L , Dreyfuss G . RNA and disease. Cell. 2009;136(4):777‐793. doi:10.1016/j.cell.2009.02.011 19239895 PMC2866189

[22] Bartel DP . MicroRNAs: target recognition and regulatory functions. Cell. 2009;136(2):215‐233. doi:10.1016/j.cell.2009.01.002 19167326 PMC3794896

[23] Bartel DP . MicroRNAs: genomics, biogenesis, mechanism, and function. Cell. 2004;116(2):281‐297. doi:10.1016/S0092-8674(04)00045-5 14744438

[24] Kopp F , Mendell JT . Functional classification and experimental dissection of long noncoding RNAs. Cell. 2018;172(3):393‐407. doi:10.1016/j.cell.2018.01.011 29373828 PMC5978744

[25] Zhang Y , Liu Y , Liu H , Tang WH . Exosomes: biogenesis, biologic function and clinical potential. Cell Biosci. 2019;9(1):1‐18. doi:10.1186/s13578-019-0282-2 30815248 PMC6377728

[26] Xiao C , Rajewsky K . MicroRNA control in the immune system: basic principles. Cell. 2009;136(1):26‐36. doi:10.1016/j.cell.2008.12.027 19135886

[27] Escobar‐Morreale HF . Polycystic ovary syndrome: Definition, aetiology, diagnosis and treatment. Nat Rev Endocrinol. 2018;14(5):270‐284. doi:10.1038/nrendo.2018.24 29569621

[28] Che X , Jian F , Chen C , Liu C , Liu G , Feng W . PCOS serum‐derived exosomal miR‐27a‐5p stimulates endometrial cancer cells migration and invasion. J Mol Endocrinol. 2020;64(1):1‐12. doi:10.1530/JME-19-0159 31710594

[29] Deswal R , Dang AS . Dissecting the role of micro‐RNAs as a diagnostic marker for polycystic ovary syndrome: a systematic review and meta‐analysis. Fertil Steril. 2020;113(3):661‐669.e2. doi:10.1016/j.fertnstert.2019.11.001 32192599

[30] Zeng Z , Lin X , Xia T , Liu W , Tian X , Li M . Identification of crucial lncRNAs, miRNAs, mRNAs, and potential therapeutic compounds for polycystic ovary syndrome by bioinformatics analysis. Biomed Res Int. 2020;2020:1–16. doi:10.1155/2020/1817094 PMC7666708 33224973

[31] Zhao C , Zhou Y , Shen X , et al. Circular RNA expression profiling in the fetal side of placenta from maternal polycystic ovary syndrome and circ_0023942 inhibits the proliferation of human ovarian granulosa cell. Arch Gynecol Obstet. 2020;301(4):963‐971. doi:10.1007/s00404-020-05495-5 32193602

[32] Yao L , Li M , Hu J , Wang W , Gao M . MiRNA‐335‐5p negatively regulates granulosa cell proliferation via SGK3 in PCOS. Reproduction. 2018;156(5):439‐444. doi:10.1530/REP-18-0229 30328340

[33] Mao Z , Fan L , Yu Q , et al. Abnormality of Klotho signaling is involved in polycystic ovary syndrome. Reprod Sci. 2018;25(3):372‐383. doi:10.1177/1933719117715129 28673204

[34] Gao L , Wu D , Wu Y , et al. MiR‐3940‐5p promotes granulosa cell proliferation through targeting KCNA5 in polycystic ovarian syndrome. Biochem Biophys Res Commun. 2020;524(4):791‐797. doi:10.1016/j.bbrc.2020.01.046 32019676

[35] Sharma M , Barai RS , Kundu I , Bhaye S , Pokar K , Idicula‐Thomas S . PCOSKBR2: a database of genes, diseases, pathways, and networks associated with polycystic ovary syndrome. Sci Rep. 2020;10(1):1‐11. doi:10.1038/s41598-020-71418-8 32895427 PMC7477240

[36] Zhang D , Tang HY , Tan L , Zhao DM . MALAT1 is involved in the pathophysiological process of PCOS by modulating TGFβ signaling in granulosa cells. Mol Cell Endocrinol. 2019;2020(499):1‐7. doi:10.1016/j.mce.2019.110589 31557499

[37] Huang X , Wu B , Chen M , et al. Depletion of exosomal circLDLR in follicle fluid derepresses miR‐1294 function and inhibits estradiol production via CYP19A1 in polycystic ovary syndrome. Aging (Albany NY). 2020;12(15):15414‐15435. doi:10.18632/AGING.103602 32651991 PMC7467373

[38] Ma Y , Ma L , Cao Y , Zhai J . Construction of a ceRNA‐based lncRNA‐mRNA network to identify functional lncRNAs in polycystic ovarian syndrome. Aging (Albany NY). 2021;13(6):8481‐8496. doi:10.18632/aging.202659 33714202 PMC8034915

[39] Udesen PB , Glintborg D , Sørensen AE , et al. Metformin decreases mir‐122, mir‐223 and mir‐29a in women with polycystic ovary syndrome. Endocr Connect. 2020;9(11):1075‐1084. doi:10.1530/EC-20-0195 33112812 PMC7774773

[40] Jiang X , Li J , Zhang B , et al. Differential expression profile of plasma exosomal microRNAs in women with polycystic ovary syndrome. Fertil Steril. 2021;115(3):782‐792. doi:10.1016/j.fertnstert.2020.08.019 33041053

[41] Wang L‐P , Peng X‐Y , Lv X‐Q , et al. High throughput circRNAs sequencing profile of follicle fluid exosomes of polycystic ovary syndrome patients. J Cell Physiol. 2019;234(9):15537‐15547. doi:10.1002/jcp.28201 30779115

[42] Hu J , Tang T , Zeng Z , Wu J , Tan X , Yan J . The expression of small RNAs in exosomes of follicular fluid altered in human polycystic ovarian syndrome. PeerJ. 2020;2020(2):e8640. doi:10.7717/peerj.8640 PMC7035867 32117643

[43] Cui X , Sun X , Niu W , et al. Long non‐coding RNA: potential diagnostic and therapeutic biomarker for major depressive disorder. Med Sci Monit. 2016;22:5240‐5248. doi:10.12659/MSM.899372 28039689 PMC5221417

[44] Huang X , Pan J , Wu B , Teng X . Construction and analysis of a lncRNA (PWRN2)‐mediated ceRNA network reveal its potential roles in oocyte nuclear maturation of patients with PCOS. Reprod Biol Endocrinol. 2018;16(1):1‐13. doi:10.1186/s12958-018-0392-4 30075721 PMC6091030

[45] Wang W , Ji J , Li J , et al. Several critical genes and microRNAs associated with the development of polycystic ovary syndrome. Ann Endocrinol (Paris). 2020;81(1):18‐27. doi:10.1016/j.ando.2019.10.002 32127169

[46] Fu L‐L , Xu Y , Li D‐D , et al. Expression profiles of mRNA and long noncoding RNA in the ovaries of letrozole‐induced polycystic ovary syndrome rat model through deep sequencing. Gene. 2018;657(2017):19‐29. doi:10.1016/j.gene.2018.03.002 29505837

[47] Huang J , Zhao J , Geng X , et al. Long non‐coding RNA lnc‐CCNL1‐3:1 promotes granulosa cell apoptosis and suppresses glucose uptake in women with polycystic ovary syndrome. Mol Ther Nucleic Acids. 2021;23(March):614‐628. doi:10.1016/j.omtn.2020.12.008 33552682 PMC7819816

[48] Memczak S , Jens M , Elefsinioti A , et al. Circular RNAs are a large class of animal RNAs with regulatory potency. Nature. 2013;495(7441):333‐338. doi:10.1038/nature11928 23446348

[49] Che Q , Liu M , Xu J , et al. Characterization of circular RNA expression profiles in cumulus cells from patients with polycystic ovary syndrome. Fertil Steril. 2019;111(6):1243‐1251.e1. doi:10.1016/j.fertnstert.2019.02.023 30979425

[50] Ma Z , Zhao H , Zhang Y , Liu X , Hao C . Novel circular RNA expression in the cumulus cells of patients with polycystic ovary syndrome. Arch Gynecol Obstet. 2019;299(6):1715‐1725. doi:10.1007/s00404-019-05122-y 30941555

[51] Lu J , Xue Y , Wang Y , et al. CiRS‐126 inhibits proliferation of ovarian granulosa cells through targeting the miR‐21‐PDCD4‐ROS axis in a polycystic ovarian syndrome model. Cell Tissue Res. 2020;381(1):189‐201. doi:10.1007/s00441-020-03187-9 32468088

[52] Deng L , Chen Q , Xie J , Wei W , Hui H . circPUM1 promotes polycystic ovary syndrome progression by sponging to miR‐760. Gene. 2020;754:144903. doi:10.1016/j.gene.2020.144903 32540374

[53] Sun X , Yan X , Liu K , et al. Lncrna h19 acts as a cerna to regulate the expression of ctgf by targeting mir‐19b in polycystic ovary syndrome. Brazilian J Med Biol Res. 2020;53(11):1‐7. doi:10.1590/1414-431x20209266 PMC7552896 33053114

[54] Wang C , Yue S , Jiang Y , et al. LncRNA GAS5 is upregulated in polycystic ovary syndrome and regulates cell apoptosis and the expression of IL‐6. J Ovarian Res. 2020;13(1):1‐8. doi:10.1186/s13048-020-00748-y PMC7733246 33308258

[55] Hocaoglu M , Demirer S , Loclar Karaalp I , et al. Identification of miR‐16‐5p and miR‐155‐5p microRNAs differentially expressed in circulating leukocytes of pregnant women with polycystic ovary syndrome and gestational diabetes. Gynecol Endocrinol. 2021;37(3):216‐220. doi:10.1080/09513590.2020.1843620 33148068

[56] Arancio W , Calogero Amato M , Magliozzo M , Pizzolanti G , Vesco R , Giordano C . Serum miRNAs in women affected by hyperandrogenic polycystic ovary syndrome: the potential role of miR‐155 as a biomarker for monitoring the estroprogestinic treatment. Gynecol Endocrinol. 2018;34(8):704‐708. doi:10.1080/09513590.2018.1428299 29385860

[57] Ebrahimi SO , Reiisi S , Parchami BS . Increased risk of polycystic ovary syndrome (PCOS) associated with CC genotype of miR‐146a gene variation. Gynecol Endocrinol. 2018;34(9):793‐797. doi:10.1080/09513590.2018.1460341 29637801

[58] Dehghan Z , Mohammadi‐Yeganeh S , Salehi M . MiRNA‐155 regulates cumulus cells function, oocyte maturation, and blastocyst formation. Biol Reprod. 2020;103(3):548‐559. doi:10.1093/biolre/ioaa098 32507875

[59] Ghasemi M , Heidari Nia M , Hashemi M , et al. An association study of polymorphisms in the H19 imprinted gene in an Iranian population with the risk of polycystic ovary syndrome. Biol Reprod. 2020;103(5):978‐985. doi:10.1093/biolre/ioaa131 32720692

[60] Yu J , Ding C , Guan S , Wang C . Association of single nucleotide polymorphisms in the RAB5B gene 3UTR region with polycystic ovary syndrome in Chinese Han women. Biosci Rep. 2019;39(5):1‐13. doi:10.1042/BSR20190292 PMC6522744 31036605

[61] Díaz M , Bassols J , López‐Bermejo A , de Zegher F , Ibáñez L . Low Circulating Levels of miR‐451a in Girls with Polycystic Ovary Syndrome: Different Effects of Randomized Treatments. J Clin Endocrinol Metab. 2020;105(3):e273‐e281. doi:10.1210/clinem/dgz204 31730174

[62] Ding Y , He P , Li Z . MicroRNA‐9119 regulates cell viability of granulosa cells in polycystic ovarian syndrome via mediating Dicer expression. Mol Cell Biochem. 2020;465(1–2):187‐197. doi:10.1007/s11010-019-03678-6 31894528

[63] Mcallister JM , Han AX , Modi BP , et al. MiRNA profiling reveals miRNA‐130b‐3p mediates DENND1A variant 2 expression and androgen biosynthesis. Endocrinology. 2019;160(8):1964‐1981. doi:10.1210/en.2019-00013 31184707 PMC6656421

[64] Cirillo F , Catellani C , Lazzeroni P , et al. MiRNAs regulating insulin sensitivity are dysregulated in polycystic ovary syndrome (PCOS) ovaries and are associated with markers of inflammation and insulin sensitivity. Front Endocrinol (Lausanne). 2019;10(December):1‐8. doi:10.3389/fendo.2019.00879 31920988 PMC6923204

[65] Butler AE , Ramachandran V , Cunningham TK , et al. Increased microRNA levels in women with polycystic ovarian syndrome but without insulin resistance: a pilot prospective study. Front Endocrinol (Lausanne). 2020;11(September):1‐7. doi:10.3389/fendo.2020.571357 33101204 PMC7556216

[66] Sørensen AE , Udesen PB , Maciag G , et al. Hyperandrogenism and metabolic syndrome are associated with changes in serum‐derived microRNAs in women with polycystic ovary syndrome. Front Med. 2019;6(November):1‐13. doi:10.3389/fmed.2019.00242 PMC6839444 31737638

[67] Lionett S , Kiel IA , Camera DM , et al. Circulating and adipose tissue miRNAs in women with polycystic ovary syndrome and responses to high‐intensity interval training. Front Physiol. 2020;11(July):1‐13. doi:10.3389/fphys.2020.00904 32848854 PMC7406716

[68] Rooda I , Hasan MM , Roos K , et al. Cellular, extracellular and extracellular vesicular miRNA profiles of pre‐ovulatory follicles indicate signaling disturbances in polycystic ovaries. Int J Mol Sci. 2020;21(24):1‐23. doi:10.3390/ijms21249550 PMC7765449 33333986

[69] Luo H , Han Y , Liu J , Zhang Y . Identification of microRNAs in granulosa cells from patients with different levels of ovarian reserve function and the potential regulatory function of miR‐23a in granulosa cell apoptosis. Gene. 2019;686:250‐260. doi:10.1016/j.gene.2018.11.025 30453069

[70] Song Y , Yu G , Xiang Y , Li Y , Wan L , Tan L . Altered miR‐186 and miR‐135a contribute to granulosa cell dysfunction by targeting ESR2: a possible role in polycystic ovary syndrome. Mol Cell Endocrinol. 2019;494:110478. doi:10.1016/j.mce.2019.110478 31173821

[71] Xue Y , Lv J , Xu P , et al. Identification of microRNAs and genes associated with hyperandrogenism in the follicular fluid of women with polycystic ovary syndrome. J Cell Biochem. 2018;119(5):3913‐3921. doi:10.1002/jcb.26531 29193229

[72] Xia H , Zhao Y . miR‐155 is high‐expressed in polycystic ovarian syndrome and promotes cell proliferation and migration through targeting PDCD4 in KGN cells. Artif Cells, Nanomedicine Biotechnol. 2020;48(1):197‐205. doi:10.1080/21691401.2019.1699826 31851829

[73] Hou Y , Wang Y , Xu S , Qi G , Wu X . Bioinformatics identification of microRNAs involved in polycystic ovary syndrome based on microarray data. Mol Med Rep. 2019;20(1):281‐291. doi:10.3892/mmr.2019.10253 31115532 PMC6579986

[74] Li Y , Xiang Y , Song Y , Wan L , Yu G , Tan L . Dysregulated miR‐142,‐33b and‐423 in granulosa cells target TGFBR1 and SMAD7: a possible role in polycystic ovary syndrome. Mol Hum Reprod. 2019;25(10):638‐646. 30865275 10.1093/molehr/gaz014

[75] O’Doherty AM , O’Brien YM , Browne JA , Wingfield M , O’Shea LC . Expression of granulosa cell microRNAs, AVEN and ATRX are associated with human blastocyst development. Mol Reprod Dev. 2018;85(11):836‐848. doi:10.1002/mrd.22990 29693772

[76] Nanda D , Chandrasekaran SP , Ramachandran V , Kalaivanan K , Carani VA . Evaluation of serum miRNA‐24, miRNA‐29a and miRNA‐502‐3p expression in PCOS subjects: correlation with biochemical parameters related to PCOS and insulin resistance. Indian J Clin Biochem. 2020;35(2):169‐178. doi:10.1007/s12291-018-0808-0 32226248 PMC7093609

[77] Geng Y , Sui C , Xun Y , Lai Q , Jin L . MiRNA‐99a can regulate proliferation and apoptosis of human granulosa cells via targeting IGF‐1R in polycystic ovary syndrome. J Assist Reprod Genet. 2019;36(2):211‐221. doi:10.1007/s10815-018-1335-x 30374732 PMC6420594

[78] Rashad NM , Ateya MAM , Saraya YS , et al. Association of miRNA ‐ 320 expression level and its target gene endothelin‐1 with the susceptibility and clinical features of polycystic ovary syndrome. J Ovarian Res. 2019;12(1):1‐10. doi:10.1186/s13048-019-0513-5 31064393 PMC6505291

[79] Xiong Z , Li B , Wang W , et al. MiR‐140 targets RAP2A to enable the proliferation of insulin‐treated ovarian granulosa cells. J Ovarian Res. 2020;13(1):1‐11. doi:10.1186/s13048-020-0611-4 PMC7003402 32024547

[80] Butler AE , Ramachandran V , Hayat S , et al. Expression of microRNA in follicular fluid in women with and without PCOS. Sci Rep. 2019;9(1):1‐9. doi:10.1038/s41598-019-52856-5 31705013 PMC6841741

[81] Tabrizi ZPF , Miraj S , Tahmasebian S , Ghasemi S . Plasma Levels of miR‐27a, miR‐130b, and miR‐301a in polycystic ovary syndrome. Int J Mol Cell Med. 2020;9(3):198‐206. doi:10.22088/IJMCM.BUMS.9.3.198 33274182 PMC7703662

[82] Butler AE , Hayat S , Dargham SR , et al. Alterations in long noncoding RNAs in women with and without polycystic ovarian syndrome. Clin Endocrinol (Oxf). 2019;91(6):793‐797. doi:10.1111/cen.14087 31482638

[83] Tan J , Hao XL , Zhao TT , Ying JL , Li T , Cheng L . Association between long‐chain non‐coding RNA SRA1 gene single‐nucleotide polymorphism and polycystic ovary syndrome susceptibility. J Assist Reprod Genet. 2020;37(10):2513‐2523. doi:10.1007/s10815-020-01922-3 32783135 PMC7550517

[84] Jiao J , Shi B , Wang T , et al. Characterization of long non‐coding RNA and messenger RNA profiles in follicular fluid from mature and immature ovarian follicles of healthy women and women with polycystic ovary syndrome. Hum Reprod. 2018;33(9):1735‐1748. doi:10.1093/humrep/dey255 30052945

[85] Fawzy MS , Abdelghany AA , Toraih EA , Mohamed AM . Circulating long noncoding RNAS H19 and GAS5 are associated with type 2 diabetes but not with diabetic retinopathy: a preliminary study. Bosn J Basic Med Sci. 2020;20(3):365‐371. doi:10.17305/bjbms.2019.4533 31999937 PMC7416173

[86] Bouckenheimer J , Fauque P , Lecellier CH , et al. Differential long non‐coding RNA expression profiles in human oocytes and cumulus cells. Sci Rep. 2018;8(1):1‐13. doi:10.1038/s41598-018-20727-0 29396444 PMC5797088

[87] Jiang B , Xue M , Xu D , Song J , Zhu S . Down‐regulated lncRNA HOTAIR alleviates polycystic ovaries syndrome in rats by reducing expression of insulin‐like growth factor 1 via microRNA‐130a. J Cell Mol Med. 2020;24(1):451‐464. doi:10.1111/jcmm.14753 31733099 PMC6933321

[88] Zhen J , Li J , Li X , et al. Downregulating lncRNA NEAT1 induces proliferation and represses apoptosis of ovarian granulosa cells in polycystic ovary syndrome via microRNA‐381/IGF1 axis. J Biomed Sci. 2021;28(1):1‐14. 34266430 10.1186/s12929-021-00749-z PMC8281489

[89] Wu G , Yang Z , Chen Y , Li X , Yang J , Yin T . Downregulation of Lnc‐OC1 attenuates the pathogenesis of polycystic ovary syndrome. Mol Cell Endocrinol. 2020;506(February):110760. doi:10.1016/j.mce.2020.110760 32070768

[90] Lin H , Xing W , Li Y , Xie Y , Tang X , Zhang Q . Downregulation of serum long noncoding RNA GAS5 may contribute to insulin resistance in PCOS patients. Gynecol Endocrinol. 2018;34(9):784‐788. doi:10.1080/09513590.2018.1459548 29648472

[91] Liu M , Zhu H , Li Y , Zhuang J , Cao T , Wang Y . Expression of serum lncRNA‐Xist in patients with polycystic ovary syndrome and its relationship with pregnancy outcome. Taiwan J Obstet Gynecol. 2020;59(3):372‐376. doi:10.1016/j.tjog.2020.03.006 32416882

[92] Wang L , Fan H , Zou Y , et al. Aberrant expression of long non‐coding RNAs in exosomes in follicle fluid from PCOS patients. Front Genet. 2021;11(February):1‐10. doi:10.3389/fgene.2020.608178 PMC7925891 33679867

[93] Zhang X , Xu Y , Fu L , et al. Identification of mRNAs related to endometrium function regulated by lncRNA CD36‐005 in rat endometrial stromal cells. Reprod Biol Endocrinol. 2018;16(1):1‐11. doi:10.1186/s12958-018-0412-4 30322386 PMC6190555

[94] Geng X , Zhao J , Huang J , et al. lnc‐MAP3K13‐7:1 inhibits ovarian GC proliferation in PCOS via DNMT1 downregulation‐mediated CDKN1A promoter hypomethylation. Mol Ther. 2021;29(3):1279‐1293. doi:10.1016/j.ymthe.2020.11.018 33212300 PMC7934583

[95] Yang R , Chen J , Wang L , Deng A . LncRNA BANCR participates in polycystic ovary syndrome by promoting cell apoptosis. Mol Med Rep. 2019;19(3):1581‐1586. doi:10.3892/mmr.2018.9793 30592281 PMC6390073

[96] Chen Y , Zhang X , An Y , Liu B , Lu M . LncRNA HCP5 promotes cell proliferation and inhibits apoptosis via miR‐27a‐3p/IGF‐1 axis in human granulosa‐like tumor cell line KGN. Mol Cell Endocrinol. 2019;2020(503):110697. doi:10.1016/j.mce.2019.110697 31891769

[97] Guo H , Li T , Sun X . LncRNA HOTAIRM1, miR‐433‐5p and PIK3CD function as a ceRNA network to exacerbate the development of PCOS. J Ovarian Res. 2021;14(1):1‐9. doi:10.1186/s13048-020-00742-4 33485372 PMC7827980

[98] Han Q , Zhang W , Meng J , Ma L , Li A . LncRNA‐LET inhibits cell viability, migration and EMT while induces apoptosis by up‐regulation of TIMP2 in human granulosa‐like tumor cell line KGN. Biomed Pharmacother. 2018;100(1677):250‐256. doi:10.1016/j.biopha.2018.01.162 29432996

[99] Liu G , Liu S , Xing G , Wang F . lncRNA PVT1/microRNA‐17‐5p/PTEN axis regulates secretion of E2 and P4, proliferation, and apoptosis of ovarian granulosa cells in PCOS. Mol Ther ‐ Nucleic Acids. 2020;20(288):205‐216. doi:10.1016/j.omtn.2020.02.007 32179451 PMC7078124

[100] Butler AE , Hayat S , Dargham SR , et al. Long non‐coding RNA expression in non‐obese women with polycystic ovary syndrome and weight‐matched controls. Reprod Biomed Online. 2020;41(4):579‐583. doi:10.1016/j.rbmo.2020.06.018 32819839

[101] Qin L , Huang CC , Yan XM , Wang Y , Li ZY , Wei XC . Long non‐coding RNA h19 is associated with polycystic ovary syndrome in Chinese women: a preliminary study. Endocr J. 2019;66(7):587‐595. doi:10.1507/endocrj.EJ19-0004 30982795

[102] Zhao J , Xu J , Wang W , et al. Long non‐coding RNA LINC‐01572:28 inhibits granulosa cell growth via a decrease in p27 (Kip1) degradation in patients with polycystic ovary syndrome. EBioMedicine. 2018;36:526‐538. doi:10.1016/j.ebiom.2018.09.043 30293818 PMC6197751

[103] Sang X . Withdrawal: “Long Non‐coding RNA NEAT1 Drives the Development of Polycystic Ovary Syndrome via Sponging Multiple MicroRNAs” by Xia Sang and Yuzhen Zhang. Cell Biol Int. 2020;0–1. doi:10.1002/cbin.11349 32222120

[104] Youssef HMG , Marei ES , Rashed LA . Long non‐coding RNA steroid receptor activator in polycystic ovary syndrome : possible association with metabolic syndrome. 2019. doi:10.12891/ceog4774.2019

[105] Li Y , Zhang J , Liu YD , et al. Long non‐coding RNA TUG1 and its molecular mechanisms in polycystic ovary syndrome. RNA Biol. 2020;17(12):1798‐1810. doi:10.1080/15476286.2020.1783850 32559120 PMC7714456

[106] Che Q , Liu M , Zhang D , et al. Long noncoding RNA HUPCOS promotes follicular fluid androgen excess in PCOS patients via aromatase inhibition. J Clin Endocrinol Metab. 2020;105(4):1086‐1097. doi:10.1210/clinem/dgaa060 32016412

[107] Wang Q , Shang J , Zhang Y , Zhou W . Metformin and sitagliptin combination therapy ameliorates polycystic ovary syndrome with insulin resistance through upregulation of lncRNA H19. Cell Cycle. 2019;18(19):2538‐2549. doi:10.1080/15384101.2019.1652036 31405334 PMC6738908

[108] Zhao J , Huang J , Geng X , et al. Polycystic ovary syndrome: Novel and hub lncRNAs in the insulin resistance‐associated lncRNA–mRNA network. Front Genet. 2019;10:1‐12. doi:10.3389/fgene.2019.00772 31507635 PMC6715451

[109] Jin L , Yang Q , Zhou C , et al. Profiles for long non‐coding RNAs in ovarian granulosa cells from women with PCOS with or without hyperandrogenism. Reprod Biomed Online. 2018;37(5):613‐623. doi:10.1016/j.rbmo.2018.08.005 30224242

[110] Gao H , Jiang J , Shi Y , Chen J , Zhao L , Wang C . The LINC00477/miR‐128 axis promotes the progression of polycystic ovary syndrome by regulating ovarian granulosa cell proliferation and apoptosis. Reprod Biol Endocrinol. 2021;19(1):1‐8. doi:10.1186/s12958-021-00718-z 33622342 PMC7901218

[111] Li Y , Wang H , Zhou D , Shuang T , Zhao H , Chen B . Up‐regulation of long noncoding RNA SRA promotes cell growth, inhibits cell apoptosis, and induces secretion of estradiol and progesterone in ovarian granular cells of mice. Med Sci Monit. 2018;24:2384‐2390. doi:10.12659/MSM.907138 29674607 PMC5928913

[112] Li L , Zhu J , Ye F , et al. Upregulation of the lncRNA SRLR in polycystic ovary syndrome regulates cell apoptosis and IL‐6 expression. Cell Biochem Funct. 2020;38(7):880‐885. doi:10.1002/cbf.3507 31999854 PMC7586972

